# Hydrotherapy as a recovery strategy after exercise: a pragmatic controlled trial

**DOI:** 10.1186/1472-6882-13-180

**Published:** 2013-07-18

**Authors:** Antonio I Cuesta-Vargas, Alvaro Travé-Mesa, Alberto Vera-Cabrera, Dario Cruz-Terrón, Adelaida M Castro-Sánchez, Cesar Fernández-de-las-Peñas, Manuel Arroyo-Morales

**Affiliations:** 1 School of Clinical Science, Faculty of Health Science, Queensland University Technology, Brisbane, Australia; 2 Department of Physical Therapy, Universidad de Granada, Granada, Spain; 3 Sport Spa Club Yo10-Granada, Granada, Spain; 4 Department of Physical Therapy, Universidad de Almeria, Almeria, Spain; 5 Department of Physical Therapy, Occupational Therapy, Physical Medicine and Rehabilitation, Universidad Rey Juan Carlos, Alcorcón, Spain

**Keywords:** Hydrotherapy, Heart rate, Fatigue, Strength, Blood pressure, Body temperature

## Abstract

**Background:**

Our aim was to evaluate the recovery effects of hydrotherapy after aerobic exercise in cardiovascular, performance and perceived fatigue.

**Methods:**

A pragmatic controlled repeated measures; single-blind trial was conducted. Thirty-four recreational sportspeople visited a Sport-Centre and were assigned to a Hydrotherapy group (experimental) or rest in a bed (control) after completing a spinning session. Main outcomes measures including blood pressure, heart rate, handgrip strength, vertical jump, self-perceived fatigue, and body temperature were assessed at baseline, immediately post-exercise and post-recovery. The hypothesis of interest was the session*time interaction.

**Results:**

The analysis revealed significant session*time interactions for diastolic blood pressure (P=0.031), heart rate (P=0.041), self perceived fatigue (P=0.046), and body temperature (P=0.001); but not for vertical jump (P=0.437), handgrip (P=0.845) or systolic blood pressure (P=0.266). Post-hoc analysis revealed that hydrotherapy resulted in recovered heart rate and diastolic blood pressure similar to baseline values after the spinning session. Further, hydrotherapy resulted in decreased self-perceived fatigue after the spinning session.

**Conclusions:**

Our results support that hydrotherapy is an adequate strategy to facilitate cardiovascular recovers and perceived fatigue, but not strength, after spinning exercise.

**Trial registration:**

ClinicalTrials.gov Identifier: NCT01765387

## Background

The translation of *Salus per aqua* to a metropolitan environment is a Club Spa facility where the primary purpose is fitness by offering a variety of professionally administered hydrotherapy services on a day-use basis. Different strategies including massage [1], compression garments [2], cryotherapy [3], contrast baths [4], and electromyostimulation [5] are effective for enhancing recovery after exercise, but their effects remain to be fully elucidated. Hydrotherapy could be a strategy that may be effective for assisting in recovery after exercise. Several exercises modalities are offered in Spa Club centers for improving aerobic capacity. Spinning is considered a high intensity interval exercise modality [6] which leads to improve bone mineral density as a health benefit [7]. Furthermore, high-intensity interval exercise may improve health status [8]. Nevertheless, immediate deleterious effects with these exercise modalities include an increase of cardiac biomarkers [9], rhabdomyolysis [10], or apparition of side effects in illness population [11].

It seems that water immersion induces cardiovascular system response mediated by parasympathetic branches of autonomic nervous system [12]. This response induces a cardio-protector effect characterized by bradycardia and reduction of cardiac output [13]. It is not known if a similar response is elicited during the recovery phase after exercise.

Preliminary research has demonstrated that contrast bath therapy, a commonly used modality in sports centers, is associated with faster recovery of power production during a jump squat test [14]. However, when exercise involves isometric performance the contrast bath therapy was associated in reduced performance [14]. Although the effects of hydrotherapy are controversial, the beneficial effects on self-rated recovery may further support the use of these recovery strategies [15]. Research focusing on therapeutic recovery approaches e.g., hydrotherapy on physiological and performance parameters following exercise is scare.

Better knowledge relative to the appropriate combination of exercise and hydrotherapy as a therapeutic approach to generate wellness could improve upon the effects of combined strategies frequently used in sports centers. Therefore, the aim of this study was to evaluate the effects of hydrotherapy on recovery following a spinning session focusing on cardiovascular, muscle performance and self-perceived fatigue in group of recreational sportspeople.

## Methods

This was a pragmatic, controlled, simple-blind study investigating the immediate effects of hydrotherapy (experimental group) or rest in a supine position (control) after a spinning exercise in recreational sportspeople. Both groups performed the recovery session (hydrotherapy or rest in supine position) after completing the exercise protocol. Participants were allocated to the control or intervention group based on their order of arrival. Because special facilities were required (hydrotherapy and spinning equipment), we contacted the Y010 Sport Club (Granada, Spain) to carry out the study. We had an ethical obligation with the Y010 Sport Centre to avoid modifying the preferences of the club users with respect to hydrotherapy, which did not permit us randomization. During the first 2 months, we recruited the intervention group and in the subsequent 3 months the control group.

### Subjects

Potentially eligible participants were initially screened for the inclusion criteria including: 5–10 hours/wk of physical activity, no pharmaceutical drug intake in the past 3 months, no use of tobacco or other addictive substances, no signs or symptoms of medical disease, no pain symptoms in the previous 12 months, and no contraindication for high-intensity exercise and participation in previous studies about recovery after exercise [1]. Exclusion criteria including: practice sport in professional setting / to be affected by orthopaedic or general conditions as diabetes or hypertension. After this initial session, participants arrived at the sport-centre between 17:00–21:00 hours on 2 separate occasions to avoid circadian rhythm-induced variations There was a 1-week interval period between each treatment session. Participants were requested to abstain from caffeine, alcohol, food and exercise for 24 h prior to starting the study to reduce the influence of these substances on outcomes. The exercise session was supervised by a certified trainer with 10 year of experience in Spinning. Informed consent was obtained from all subjects, and study procedures were consistent with the Helsinki declaration. The study was approved by University of Granada Ethic Commmittee.

### Exercise session

The spinning session was performed using the same protocol for all participants after a 24 h physical rest as suggested [16]. Participants were asked to perform this session as a regular spinning class. The session was carried out in the afternoon (room temperature 22±3.5°C) on a modified spinning bike (Keiser®, M3). They were allowed to drink water during the session and completed the spinning session while listening to a compilation of music that lasted 50 min and was composed of 9 tracks.

Each track corresponded to a specific phase of the spinning session. The phases were labeled as warm-up, sitting, seated climbing, jumping, and running, based on the official spinning program manual [17]. Some phases were repeated during the session, the compilation being purposely designed for beginners in a spinning class. In addition to the music protocol, participants were asked to maintain a pedal stroke cadence that had previously been established for each track. For the resistance applied to the flywheel, they were free to adjust it according to their sensation and interpretation of the spinning session.

### Recovery procedures

#### Experimental group

A cycle of 3 Vichy shower and whirlpool baths were applied during 30-minutes period. Vichy sedative shower was applied for 90–120 sec to the sides of the trunk and the abdomen, avoiding as much as possible the gall bladder area, at a temperature of 36-38°C. A short, partial jet spray followed the shower. A whirlpool bath was administered where subjects immersed the body until their clavicle level for a 10min period with a water temperature ranging 33.5-35.5°C. Aromatherapy application using lavender and chamomile oils was used in all hydrotherapy sessions.

#### Control group

The control group performed a rest session in supine position in a room with neutral temperature condition with a same duration to hydrotherapy session. Participants of both groups were encouraged to drink water “*ad libitum*” to prevent dehydration.

### Measurements

Outcomes were obtained at baseline, after each exercise intervention, and at the end of the recovery session (hydrotherapy, rest). Self-rated measurements, physiological and performance parameters were included in this study with the intention to have a broad and practical view of different aspect of recovery after fatigue.

#### Blood pressure/heart rate measurements

An Omron HEM-737 validated automatic oscillometric device (Kyoto, Japan) was used for assessing blood pressure and heart rate measurements. Measurements were performed in triplicate and the average was used for data analysis.

#### Upper body muscular strength

Handgrip strength was assessed bilaterally with a digital dynamometer (TKK 5101 Grip-D; Takey, Tokyo, Japan) as previously described [18]. Subjects performed the test twice, allowing a 3-minute rest period between measures. The mean value of 2 trials was scored. This test has been shown to be valid and reliable [19].

#### Lower body muscular strength

Vertical jump performance was assessed with a squat jump with infrared photocell mat (Ergo-jump Globus, Codogne, Italy) [20]. The jumps were performed with hands held on the hips and attaining 90° knee flexion at the start of the push-off phase. Participants performed 3 trials of each jump, and the best attempt was retained for the analysis.

*A Visual Analogue Fatigue Scale (VAFS)* was used to assess the intensity of fatigue. The visual analogue scale is a 100 mm line anchored with 0 at one representing no fatigue, and 100 at the other end representing the worst fatigue imaginable. Subjects were asked to mark on the line the point that they feel their perception of their current fatigue state. The score was obtained by measuring the line from “No Fatigue” to the point indicated by the subject that represents their fatigue level: the higher the VAFS score, the higher the fatigue.

#### Body temperature

We assessed body temperature using the OMRON GentleTemp 510, an infrared thermometry device (MC-510-E2, Kyoto, Japan). The temperature of the room ranged between 23 and 26°.

### Sample size

According a pilot study, a priori sample size calculation indicated 14 patients per group were required to detect a significant difference of 20% in fatigue analogue scale between the intervention and control group (Effect size d =1, alpha=0.05, beta=0.08).

### Statistical analysis

Data were analyzed using the SPSS package (version 19.0). Mean and standard deviations or 95% confidence intervals of the values were calculated for each variable. The Kolmogorov-Smirnov test showed a normal distribution of the data (P > 0.05). Pre-intervention values prior to each condition were compared using the independent t-tests for continuous data. A 2x3 mixed model ANOVA with session (hydrotherapy, control rest) as the between-subjects variable and time (pre-; post-exercise; post-recovery) as the within-subjects variable was used to determine the potential effects of the recovery method on each variable. Separate ANCOVAs were performed with each dependent variable. The hypothesis of interest was session * time interaction. The Bonferroni test was used for post hoc analysis. A P-value < 0.05 was considered statistically significant.

## Results

Finally, 17 male and 17 female recreational sportspeople (age: 29.4 ± 8.4 years; weight: 68.4 ± 11.7 kg; height: 172.5 ± 8.2 cm; body mass index: 22.9 ± 2.9) were included. Participants had engaged in physical activity for 4.2±2.7 hours/week (aerobic exercise: 3.1±1.9 hours/week; strength exercise: 1.2±1.4 hour/week). Pre-intervention scores for each variable were not significantly different between each session: systolic arterial pressure (P=0.079), diastolic arterial pressure (P=0.120), heart rate (P=0.263), handgrip (dominant side: P= 0.568; non-dominant side: P=0.734), jump test (P=0.078), fatigue analogue scale (P=0.067) and body temperature (P=0.311).

The ANCOVA revealed a significant session*time interaction for diastolic blood pressure (F=3.897; P=0.031) and heart rate (F=3.549; P=0.041), but not for systolic blood pressure (F=1.384; P=0.266) (Table 1). Pair-wise comparisons found significant decrease in diastolic blood pressure (P<0.05) and increase in heart rate (P<0.001) after spinning exercise. Diastolic blood pressure decreased (*P*<0.001) after rest in the control group as compared to baseline, whereas no change was observed after hydrotherapy (*P*=0.093). Heart rate increased (*P*=0.042) after rest in the control group as compared to baseline, whereas did not change in relation to baseline values after hydrotherapy (*P*=0.142).

**Table 1:** Comparison of blood pressure and heart rate between the groups at different time points

**Variable**	**Hydrotherapy (experimental group) n=17**	**Supine rest (control group) n=17**
**Systolic blood pressure (mm Hg)**		
Baseline	124.76 ± 9.83 (95% CI 119.70 – 129.82)	132.17 ± 17.25 (95% CI 123.30 – 141.04)
Post-spinning session	120.76 ± 9.49 (95% CI 115.88 – 125.64)*	123.41 ± 18.67 (95% CI 113.80 – 133.01)*
Post-recovery	118.82 ± 13.31 (95% CI 111.97 – 125.67)	128.17 ± 18.30 (95% CI 118.76 – 137.58)
**Diastolic blood pressure (mm Hg)**		
Baseline	76.05 ± 9.23 (95% CI 71.31 – 80.80)	81.00 ± 8.78 (95% CI 76.48 – 85.51)
Post- spinning session	71.05 ± 9.17(95% CI 66.34 – 75.77)*	75.41 ± 8.99 (95% CI 70.78 – 80.03)*
Post-recovery	71.82 ± 7.97 (95% CI 67.72 – 75.92)	71.58 ± 8.44 (95% CI 67.24 – 75.92)#
**Heart rate (beat/min)**		
Baseline	73.29 ± 10.89 (95% CI 67.69 –78.89)	78.00 ± 13.06 (95% CI 71.28 – 84.71)
Post-spinning session	100.0 ± 13.4 (95% CI 94.3 – 105.7)*	97.29 ± 15.16 (95% CI 89.49 – 105.09)*
Post-recovery	80.35 ± 16.60 (95% CI 74.0 – 86.3)#	85.94 ± 14.91 (95% CI 78.19 – 92.27)

Data are expressed as mean ± standard deviation (95% confidence interval).

* Bonferroni correction, P< 0.05, post-exercise vs. baseline value / # Bonferroni correction, P< 0.05, post-Spa therapy vs. Post-exercise value.

The ANCOVA did not reveal a significant session*time interaction for vertical jump (F=0.851; P=0.437), and handgrip force (F=0.148; P=0.863) (Table 2).

**Table 2:** Comparison of force handgrip and vertical jump between the groups at different time points

**Variable**	**Hydrotherapy (experimental group) n=17**	**Supine rest (control group) n=17**
**Vertical jump test (cm)**		
Baseline	22.94 ± 5.74 (95% CI 19.98 – 25.89)	26.96 ± 7.01 (95% CI 23.35 – 30.57)
Post-spinning session	23.55 ± 6.12 (95% CI 20.40 – 26.70)	28.60 ± 7.59 (95% CI 24.70 –30.52)
Post-recovery	23.06 ± 5.6 (95% CI 20.11 – 26.02)	27.88 ± 7.13 (95%CI 24.21 – 31.25)
**Force handgrip dominant side (Kg)**		
Baseline	36.63 ± 10.36 (95% CI 31.30 – 40.96)	38.83 ± 11.86 (95% CI 32.73 – 44.93)
Post-spinning session	37.16 ± 11.12 (95% CI 31.44 – 42.88)	38.96 ± 12.33 (95% CI 32.62 – 45.30)
Post-recovery	36.83 ± 12.08 (95% CI 30.62 – 43.04)	38.44 ± 12.89 (95% CI 31.81 – 45.07)
**Force handgrip non dominant side (Kg)**		
Baseline	34.32 ± 9.73 (95% CI 29.72 – 39.33)	35.54 ± 10.84 (95% CI 29.96 – 41.11)
Post-spinning session	34.72 ± 9.40 (95% CI 29.88 – 39.55)	35.81 ± 11.28 (95% CI 30.00 – 41.61)
Post-recovery	34.06 ±9.86 (95% CI 28.99 – 39.14)	34.82 ± 11.20 (95% CI 29.06 – 40.58)

Data are expressed as mean ± standard deviation (95% confidence interval).

**Figure 1:**
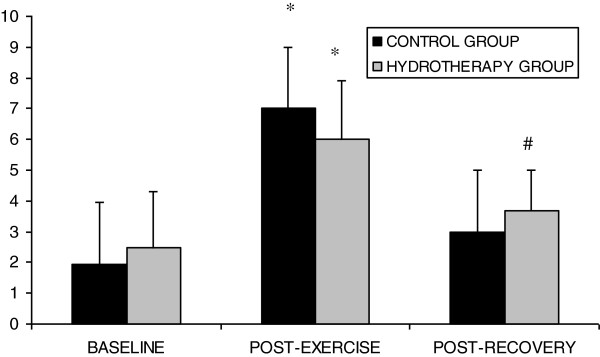
**Visual analogue scale-fatigue at different time moments of the study.** *Significant change respect baseline value (P<0.05, Bonferroni correction).

**Figure 2:**
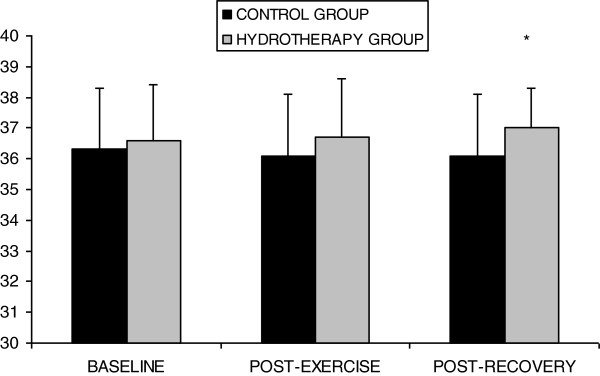
**Body temperature at different moments of the study.** *Significant change respect baseline value (P < 0.05, Bonferroni correction).

The ANOVA revealed a significant session*time interaction for fatigue (F=3.386; P=0.046) and body temperature (F=8.490; P=0.001). Pair-wise comparisons revealed that both groups exhibited significant increases in fatigue (P<0.001) after Spinning. Fatigue was no different from baseline values (P=0.436) after hydrotherapy, but was increased with respect to baseline within the control group (P=0.006) (Figure 1). A non-significant body temperature change (P>0.05) was found after spinning exercise in both groups. After hydrotherapy, body temperature was similar than baseline in control group (P=0.147), but increased in the spinning group (*P*=0.006) (Figure 2). Gender did not influence the results of these parameters.

## Discussion

The integration of hydrotherapy services combined with exercise is postulated to produce a healthier individual. A comprehensive search through different databases did not reveal any study investigating the effects of hydrotherapy as a recovery method after the application of spinning session. Our study confirms the ability of hydrotherapy to facility perceived and cardiovascular recovery of fatigue after a spinning session. However, there were no significant effects in terms of strength performance compared to rest as control strategy.

A better self-perceived recovery was obtained after the spinning session, which was developed with moderate intensity (close 70% of the maximum heart rate) eliciting a moderate perceived fatigue. These results confirm the effects of hydrotherapy after a demanding exercise results in improvements in self-perceived fatigue. The main effect of buoyancy is a reduction of post-gravitational forces that act on the musculoskeletal system allowing a greater conservation of energy that could potentially reduce perceived fatigue [12]. These results could be related to local muscle damage elicited during the spinning session [10] which may be buffered by muscle relaxation response during water immersion [21].

A relevant finding of this study was the recovery of diastolic blood pressure and heart rate to non-significant differences after hydrotherapy with respect to baseline values. Hydrotherapy resulted in recovery of the physiological state but was insufficient to reach similar cardiovascular values to baseline because of the high cardiovascular demand in the spinning session [1]. Besides, the hypotension induced by exercise [22] was buffered by the hydrotherapy session. It is suggested that immersion in water to a xiphoid level at similar water temperatures used in our study [23] produces an 11-18% heart rate decrease [24] by inducing a parasympathetic responses [1]. It is possible a summation effect of aromatherapy with lavender [25] and hydrotherapy, which may assist in heart rate recovery by promoting relaxation responses similar to other recovery strategies. These results provide relevant information about hydrotherapy since there no dangerous strain on the cardiovascular system inducing hypotension or tachycardia was promoted by water immersion [26].

Our results did not support the ability of hydrotherapy to recovery physical performance which is in agreement with previous studies [27,28]. However, there exists controversy with other treatments [12] which have shown the ability for improving physical performance. It has been proposed that the large applications of cold water as a main component of hydrotherapy can help to recover performance by inducing several cardiovascular mechanisms [12].

The current study did not support the previous reported ability of hydrotherapy to reduce body temperature [29]. The increase of body temperature after spinning is explained by a sympathetic response [30]. A thermotherapy effect was found since an increase of body temperature after hydrotherapy was observed. Thermotherapy has shown to increase tissue temperature, increase local blood flow and muscle elasticity, cause local vasodilatation, increases metabolite production, and reduce muscle spasm [31]. This thermotherapy effect may plausibly be associated with better self-perceived recovery after hydrotherapy in comparison to the control group.

Since the purpose of hydrotherapy centers is to assist people in the recovery of health [32], it is necessity to develop studies similar to the current one to determine the optimal strategies for preventing psychological and physical drop off [31].

Finally some limitations need to be recognized in this study. An immediate effect of hydrotherapy after exercise does not guarantee that these changes will be maintained during long-term follow-up periods. Therefore, more studies are needed to clarify long-term effects of hydrotherapy after different exercise modalities in an indoor setting. Second, recreational sportspeople were included in this study; it is possible that our findings would have varied in different populations. Third the lack of randomization and a small sample size warrant new studies to confirm present results.

## Conclusion

Literature about hydrotherapy used as a recovery strategy often comes from textbooks or is based on anecdotal information. Our study helps to reduce this gap in the combination of exercise and hydrotherapy using a controlled study. The findings of this study indicate that hydrotherapy showed the ability to assist with recovery of perceived fatigue and cardiovascular parameters after a spinning session but no effects on strength recovery. These improvements in the recovery profile support hydrotherapy as a practical recovery strategy. Therefore, hydrotherapy appears to be a recovery strategy that could be adopted and integrated into recovery programs for individuals participating in sport.

## Competing interests

The authors declare that they have no competing interests.

## Authors’ contributions

All the authors have made contributions to conception of this study. MAM, DCT, ATM and AVC participated in the acquisition of data. MAM, ACV, CFDLP, ACS participated in the analysis and interpretation of data and were involved in drafting the manuscript or revising it critically for important intellectual content. All the authors have given final approval of the version to be published.

## Pre-publication history

The pre-publication history for this paper can be accessed here:

http://www.biomedcentral.com/1472-6882/13/180/prepub
